# Utility of in-hospital post-delivery fasting plasma glucose to predict postpartum glucose status in women with hyperglycaemia first detected in pregnancy: A prospective cohort study

**DOI:** 10.1371/journal.pone.0239720

**Published:** 2020-10-05

**Authors:** Anneliese Wessels, Ankia Coetzee, Deidre Mason, David Hall, Mari van de Vyver, Magda Conradie

**Affiliations:** 1 Department of Medicine, Division of Endocrinology Stellenbosch University and Tygerberg Hospital, Cape Town, South Africa; 2 Department of Obstetrics & Gynaecology, Stellenbosch University and Tygerberg Hospital, Cape Town, South Africa; University of Cambridge, UNITED KINGDOM

## Abstract

**Background:**

Women with hyperglycaemia first detected in pregnancy (HFDP), including those with gestational diabetes mellitus (GDM), should undergo a glucose evaluation 4–12 weeks after delivery. Globally, suboptimal postpartum return rates limit the opportunity to intervene in women with sustained hyperglycaemia and pragmatic solutions should be sought to bridge this gap.

**Objective:**

To assess the utility of postpartum in-hospital glucose evaluation to predict the outcome of the oral glucose tolerance test (OGTT) performed 4–12 weeks after delivery.

**Methods:**

The study was performed prospectively at Tygerberg Hospital, Cape Town, South Africa. Women with HFDP, classified as GDM based on the modified National Institute for Health and Care Excellence criteria, who delivered between November 2018 and June 2019 were included in the study. Fasting plasma glucose (FPG) was performed 24–72 hours after delivery (*t1*) in the postnatal ward, provided glucose lowering medication was discontinued at delivery. An OGTT 4–12 weeks postpartum (*t2*) was scheduled for the total cohort. We compared glucose values and glucose categories at *t1* and *t2* and evaluated antenatal characteristics of women who returned, compared to the group that was lost to follow-up.

**Results:**

In-hospital post-delivery glucose assessment *(t1)* was performed in 115 women. Glucose levels were significantly lower at *t1* compared to antenatal diagnostic values (*t0)* and assessment at *t2*. Of the fourteen women with hyperglycaemia at *t2*, none had abnormal fasting glucose concentrations at *t1*. Women with HFDP who fulfilled criteria for overt diabetes at *t0*, all (24/115) had normal fasting glucose levels at *t1* except for IFG in one (1/24). The antenatal characteristics of women with HFDP who returned at *t2*, were similar to the women who did not return.

**Conclusion:**

Based on this study, in-hospital fasting glucose 24–72 hours postpartum cannot replace the OGTT 4–12 weeks postpartum. Pragmatic solutions for low postpartum return rates in women with HFDP should be pursued.

## Introduction

The burden of pre-diabetes together with Type 2 diabetes mellitus (T2DM) is rapidly increasing and is driven by the world-wide problem of obesity, urbanisation and aging [1]. Obesity and hyperglycaemia in women of reproductive age is especially important due to the associated adverse outcomes when coinciding with pregnancy. These adverse outcomes are not limited to pregnancy and the perinatal period but include the long-term risk of metabolic abnormalities in the mother and her progeny. Future abnormalities in children exposed to hyperglycaemia in pregnancy with potential intra-uterine metabolic imprinting include youth onset obesity and T2DM [2,3]_._ In turn, mothers with gestational diabetes mellitus (GDM) have a seven-fold increased risk to develop T2DM and carry this risk into subsequent pregnancies [4]. Programs to prevent and treat T2DM after hyperglycaemia first detected in pregnancy (HFDP) are of utmost importance and should follow the optimisation of glucose control during pregnancy. Continued postpartum care in women with HFDP is unfortunately limited by low follow-up rates worldwide [5].

In South-Africa, antenatal care is often the only opportunity in women of childbearing age to undergo screening for T2DM. Asymptomatic glucose abnormalities present before pregnancy are frequently first identified during these visits and categorized as GDM, a condition that is expected to resolve postpartum [6].

Globally, attempts have been made to delineate and classify abnormal glucose homeostasis in pregnancy. Prior to 2010, any form of HFDP (irrespective of the degree of hyperglycaemia) was classified as GDM. It is now recognized that GDM manifests as mild hyperglycaemia towards the third trimester and is the result of an inability of the pancreatic beta cells to compensate for the increase in insulin resistance (IR) [7]_._ GDM is confirmed with a 75-gram oral glucose tolerance test (OGTT) when the fasting plasma glucose (FPG) levels is ≥ 5.1(5.6)-6.9 mmol/L and/or 2-hour glucose values is ≥ 7.8(8.5) - 11 mmol/L depending on the diagnostic criteria used [8,9]_._ The International Association for Diabetes in Pregnancy Study Group (IADPSG) categorize HFDP with diagnostic glucose levels that meet criteria for diabetes outside of pregnancy (FPG ≥ 7 mmol/L and/or 2-hour glucose ≥ 11.1 mmol/L) as overt T2DM [9]_._ The guidelines used at Tygerberg Hospital (National Institute for Health and Care Excellence (NICE) guidelines 2015) do not provide an upper glucose limit for T2DM diagnosis in pregnancy, thus all women with HFDP are categorized as GDM [10].

Within hours after delivery, the pregnancy-related IR and hyperglycaemia as a result of GDM resolves [11,12]_._ In order to distinguish between GDM and pre-existing T2DM, all major diabetes societies advocate a glucose evaluation 4–12 weeks following delivery [9,10,13]_._ The postpartum glucose evaluation compliments antenatal glucose control, allows for timeous interventions to delay T2DM or provides an opportunity for early diagnosis and prevention of complications of pre-existing T2DM [9,10,13]_._ The timing of the 4–12 week postpartum OGTT appears to have been chosen for convenience [14], but poor attendance rates globally argues against the convenience of this visit for mothers who prioritize the care of their new-born including immunisation at this time, a problem reflected locally [15]_._ Primary health care facilities in South Africa tasked with immunization of babies at 6 weeks only offer postpartum glucose testing in the context of a clinical research setting.

Early identification of T2DM in its asymptomatic state in high risk women diagnosed with HFDP is of paramount importance, but this opportunity is thwarted by low uptake for postpartum screening [15]. Innovative and pragmatic strategies are required to bridge the gap between the need for postpartum evaluation and sub-optimal follow-up in this high risk group. The ability to identify women at highest risk for persistent glucose abnormalities while still in-hospital after delivery, would allow for improved care and efficiency within the healthcare system.

The primary aim of this study was to evaluate if FPG levels in women with HFDP obtained 24–72 hours after delivery could predict hyperglycaemia 4–12 weeks postpartum and as such identify women at highest risk of T2DM. The secondary aim was to compare antenatal characteristics of women with HFDP who returned for their 4–12 weeks postpartum visit at Tygerberg Hospital with those lost to follow-up.

## Methods

### Design and study population

The study was conducted prospectively in the postnatal wards and in the Postpartum Diabetes follow-up clinic at Tygerberg Hospital (TH), a secondary and tertiary referral centre with the largest catchment area in the Western Cape Province of South Africa. Women diagnosed with HFDP are regarded as high risk and all attend either the Obstetric Special Care or the Obstetric High-Risk clinic at TH. Deliveries are routinely planned at a gestation of 38 weeks. All HFDP women are scheduled to undergo an OGTT at the Postpartum Diabetes clinic 4–12 weeks after the index delivery. It is standard practice at our facility to discontinue glucose lowering agents just before delivery and not to re-initiate therapy if postpartum in-hospital glucose levels remain <11.1 mmol/L.

**Fig 1:**
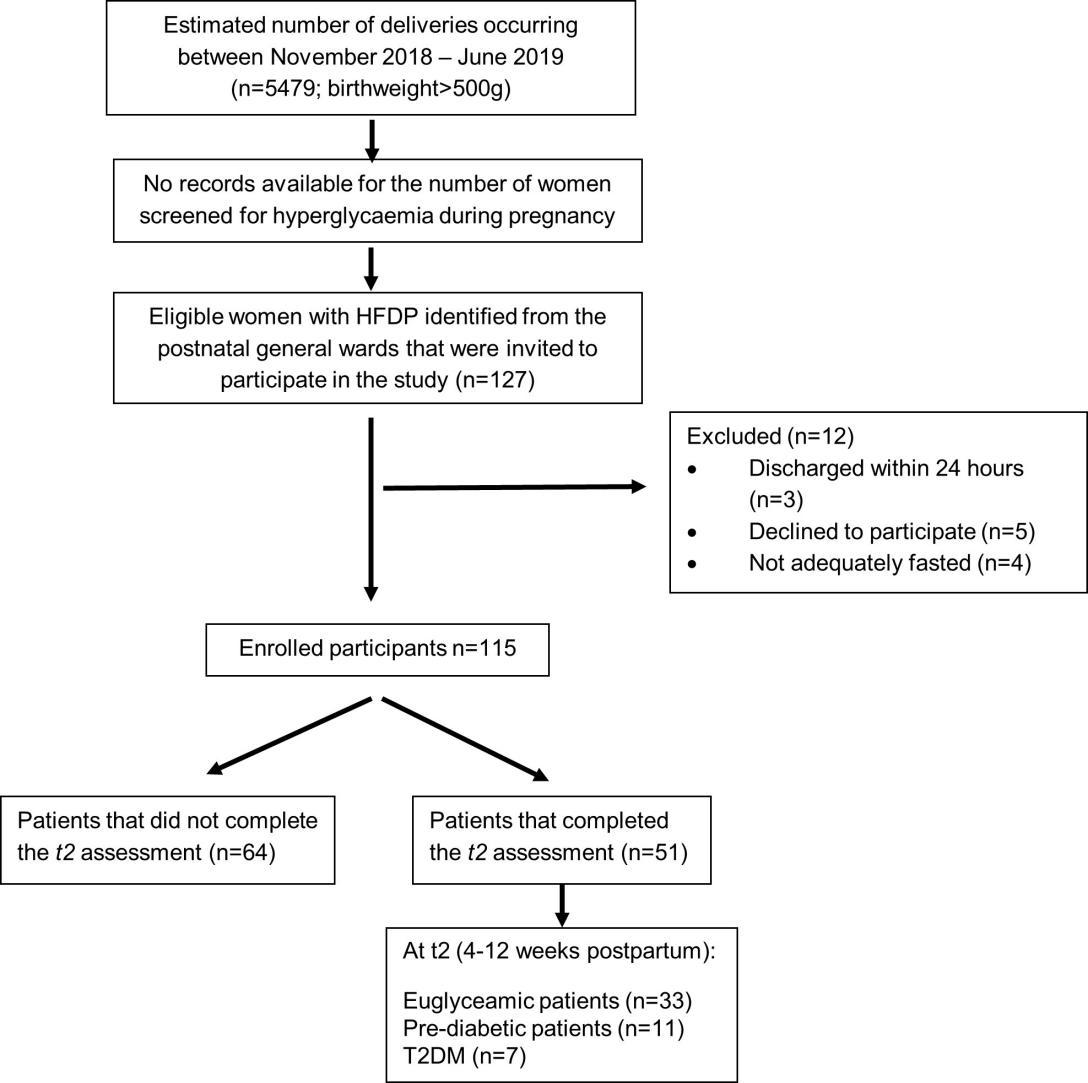
Overview of the study design. The diagram illustrates the number of eligible women that were recruited from the general postnatal ward after delivery as well as the number of enrolled patients.

Recruitment was done by the principal investigator (PI) and co-workers in the general postnatal wards within 48 hours of delivery. Women above the age of 18 years, who had HFDP and were diagnosed with GDM according to the 2015 NICE criteria [10] were included from 1 November 2018 to 6 June 2019. Women excluded from study entry included cases with overt glucose abnormalities after delivery requiring glucose lowering therapy, women unable to provide informed consent or to perform an 8 hour overnight fast and women who were discharged on the same day as delivery (Fig 1).

### Postpartum clinical and biochemical assessment

The postpartum clinical and biochemical evaluation was performed at two time points, 24 to 72 hours after delivery *(t1)* and 4–12 weeks postpartum *(t2)*. The *t1* post-delivery evaluation took place during the in-hospital stay. Antenatal data was captured from maternal hospital records and verbally during recruitment. Demographic parameters recorded included age, ethnicity, self-reported family history, socio-economic status (education, employment), anthropometry [height, weight and body mass index (BMI)], gravidity, gestational age at booking and at diagnosis, antenatal diagnostic biochemistry and the use of glucose lowering pharmacotherapy during pregnancy. The gestational age at birth-, and the mode of delivery were also documented. At *t2*, the anthropometric measurements were repeated, breastfeeding history was sought, a clinical evaluation was performed, and the antenatal data obtained were verified against institutional records.

Biochemistry at *t1* included a FPG, a concurrent capillary point-of care finger-pick glucose (POC FG) and a glycated haemoglobin (HbA1c) concentration. At *t2* the glucose evaluation consisted of an HbA1c, a 75-gram OGTT with fasting and 2-hour plasma glucose and concurrent POC tests. Biochemical measurements were performed at the National Health Laboratory Services (NHLS), a South African National Accreditation Service accredited laboratory. Plasma glucose was collected in Na fluoride tubes (Becton Dickenson, USA) and measured by means of the hexokinase method on the Roche Cobas 6000 (Roche Diagnostics, Mannheim, Germany) platform which has a measuring range from 0.11–41.6 mmol/L with a reported coefficient of variation (CV) of 1.3% at a glucose level of 5.38 mmol/L and 1.1% at a level of 13.4 mmol/L respectively. POC was utilized for the finger prick capillary glucose, and performed on the Accucheck Active (Roche Diagnostics, Mannheim, Germany) glucometer. These hand-held devices determine blood glucose concentration by means of glucose test strips and reflectance photometry and have a measuring range of 0.6–33.3mmol/L. The device is whole blood calibrated; blood glucose values displayed therefore correspond to plasma. The glucose results of both the capillary and the venous determination are reported in mmol/L. The sensitivity of POC glucose determination for the diagnosis of DM was previously reported by our group to be 96.7% (fasting) and 98.5% (2h) [16]. Both laboratory methods and the POC method have been standardized against isotope dilution mass spectrometry (ID/MS). Determination of HbA1c was based on the turbidimetric inhibition immunoassay on the Siemens ADVIA 1800 platform. The assay has a measuring range of 0.23–17.8%, with a reported CV of 1.2% at HbA1c level of 5.08% and 2.0% at a level of 10.1% respectively (15 Siemens package insert). HbA1c measurements are reported as a percentage according to the “National Glycohemoglobin Standardization Program” (NGSP) and in mmol/mol units as proposed by the International Federation of Clinical Chemistry and Laboratory Medicine (IFCC)(mmol/mol) units. The HbA1c method is traceable to the NGSP reference method.

### Ethical considerations

The study was approved by the Human Research Ethics Committee (HREC), Faculty of Medicine and Health Sciences, Stellenbosch University and Tygerberg Hospital (S18/10/223r). All of the women who participated in this study gave written informed consent.

### Statistical analysis

Statistical analysis was performed using GraphPad Prism (8.2.0). The normal distribution of data was determined using the Shapiro-Wilk and Kolmogorov-Smirnov test. Data is presented as either mean ± standard deviation (SD) (normal distribution) or median (interquartile range) (IQR) (non-parametric data). Student’s T-test with two tailed p-value, the non-parametric Mann Whitney test and Fisher’s Exact tests were performed to determine differences between the cohort of women who returned for the postpartum evaluation and those who were lost to follow up. Mixed model ANOVA with Tukey post hoc test and Chi-square analysis were performed to determine effect of group, time, group x time within the diabetes subcategories. Spearman’s correlation analysis was used to determine association between variables. Bland-Altman type and Receiver Operator Curve analysis were performed to determine the specificity and sensitivity of FPG as diagnostic predictor. Level of significance was accepted at p<0.05.

## Results

### Characteristics of total study cohort

One hundred and twenty-seven women (n = 127) eligible women that fulfilled the inclusion criteria were recruited from the general postnatal ward after delivery. Twelve women (12/127) were however excluded from the study; 3/12 were discharged within 24 hours after delivery, 5/12 did not provide consent and 4/12 were not adequately fasted. A total of one hundred and fifteen (n = 115) women were thus included in the study and underwent assessment 24–72 hours after delivery (*t1)*. Of these women, 51/115 (44.3%) attended their scheduled 4-12-week postpartum visit *(t2)*, whereas 64/115 (55.7%) were lost to follow-up (Fig 1). During the index pregnancy, the mean age of women included in this study was 33.5 ± 5.5 years with 52/115 (45.2%) being over the age of 35 years. The women self-identified as either black African (73/115 [63.5%]), mixed ancestry (36/115 [31.3%]), White (2/115 [1.7%]) or as other ethnicities (3/115 [2.6%]). Less than half (52/115 [45.2%]) of the women were formally employed and only 20/115 (17.4%) completed tertiary education. There were no differences in the baseline characteristics of participants who completed assessment at *t2* and those who did not attend the follow up visit (Table 1). Suggesting that the n = 51 women who completed the 4–12 week evaluation at *t2* is representative of the entire cohort.

**Table 1:** Baseline characteristics of total study cohort.

Parameters	Total cohort *(n = 115)*	Follow up *(n = 51)*	No follow up *(n = 64)*	p-value
**Age** (years)^a^	33.5 ± 5.5	33.5 ± 5.7	33.4 ± 5.5	n.s
≥ 35 n (%)	52 (45.2%)	24 (47.%)	28 (43.8)	n.s
**Ethnicity** n (%)				
Black African	73 (63.5%)	37 (72.5%)	36 (56.3%)	n.s
Mixed ancestry	36 (31.3%)	10 (19.6%)	26 (40.6%)	0.025*
White	2 (1.7%)	0 (0%)	2 (3.1%)	n.s
Other	3 (2.6%)	3 (5.9%)	0 (0%)	n.s
Undisclosed	1 (0.9%)	1 (1.9%)	0 (0%)	n.s
**Family history** n (%)				
First degree relative with T2DM	48 (41.7%)	19 (37.3%)	29 (45.3%)	n.s
**Previous GDM** n (%)	13 (11.3%)	5 (9.8%)	8 (12.5%)	n.s
**Level of education** n (%)				
Secondary	93 (80.9%)	41 (80.4%)	52 (81,3%)	n.s
Tertiary	20 (17.4%)	8 (15.7%)	12 (18.8%)	n.s
**Employed** n (%)	52 (45.2%)	21 (41.2%)	31 (48.4%)	n.s
**Anthropometry at booking**				
Weight (kg) ^a^	97.4 ± 21.7	97.5 ± 21.2	97.3 ± 22.2	n.s
Body mass index (BMI) (kg/m^2^)^a^	38.0 ± 8.0	37.9 ± 7.8	38 ± 8.3	n.s
BMI > 40 kg/m^2^ n (%)	47 (40.9%)	22 (43.1%)	25 (39.1%)	n.s
**Gestational age**				
Booking (weeks)^a^	13.6 ± 6.3	14.1 ± 6.1	13.2 ± 6.5	n.s
Antenatal diagnosis (weeks)^a^	27.4 ± 7.5	28.1 ± 6.6	27 ± 7.6	n.s
**HFDP type** n (%)				
GDM (NICE criteria 2015)	90 (78.3%)	37 (72.5%)	53 (82.8%)	n.s
Overt DM (IADPSG; WHO 2013)	25 (21.7%)	14 (27.5%)	11 (17.2%)	n.s
**Treatment** n (%)				
Lifestyle	36 (31.3%)	10 (19.6%)	26 (40.6%)	0.025*
Lifestyle and Metformin	73 (63.5%)	39 (76.5%)	34 (53.1%)	0.012*
Metformin and insulin	4 (3.5%)	1 (2%)	3 (5.9%)	n.s
**Delivery** n (%)				
Gestation (completed weeks)^a^	37.7 ± 1.6	37.8 ± 1.3	37.6 ± 1.8	n.s
Preterm labour	12 (7.7%)	5 (9.8%)	7 (10.9%)	n.s
Vaginal delivery	36 (31.3%)	19 (37.3%)	17 (26.6%)	n.s
Spontaneous onset of labour	13 (11.3%)	9 (17.6%)	4 (6.3%)	n.s
IOL (NVD)	52 (45.2%)	23 (45.1%)	29 (45.3%)	n.s
Elective Caesarean section	30 (26,1%)	13 (25.5)	17 (26.6)	n.s
Emergency Caesarean section	48 (41.7%)	19 (37.3)	30 (46.9)	n.s
**Macrosomia** (>4000 g) n (%)	17 (14.8%)	7(13.7%)	10 (15.6%)	n.s
**Postpartum anthropometry** (*t2*)				
Weight (kg)^a^	-	94.7 ± 20.2	-	-
Body mass index (kg/m^2^)^a^	-	37.0 ± 7.7	-	-
BMI > 40 kg/m^2^ n (%)	-	15 (29.4%)	-	-

Values are presented as either absolute values (n (%)) or mean ± SD^a^. Statistical analysis: Fisher’s exact test or ^a^Student’s T-test with two-tailed p-value.

*p<0.05 indicate significant differences between the women that returned for assessment at 4–12 weeks postpartum (n = 51) and those who were lost to follow up (n = 64). Abbreviations: IOL: induction of labour; NVD: normal vertex delivery; n.s: not significant.

The mean body mass index (BMI) of women at booking (mean gestation 13.6 ± 6.3 weeks) was 38 ± 8 kg/m^2^ with 47/115 (40.9%) of these women being morbidly obese (BMI > 40 kg/m^2^). At the postpartum assessment *(t2)*, body weight (p = 0.496) and BMI (p = 0.558) remained unchanged compared to antenatal booking values. The majority of women were multigravidas (103/115 [89.6%]), a first-degree family history of T2DM was present in 48/115 (41.7%) women and 13/103 (12.6%) had GDM in a prior pregnancy. The first antenatal booking occurred early (13.6 ± 6.3 weeks) and the diagnosis of HFDP was made after 24 weeks in 86/115 (74.8%). At HFDP diagnosis *(t0)*, 25/115 (21.7%) women had an FPG ≥ 7 mmol/L and/or a 2-hour glucose value ≥ 11.1 mmol/L and were retrospectively re-classified as overt T2DM. The remaining 90/115 (78.3%) women all had a FPG between 5.6 and 6.9 mmol/L and/or a 2-hour glucose value between 7.8 and 11 mmol/L and were thus classified as GDM. Our diagnostic criteria for GDM based on NICE 2015, differ from WHO 2013 guidelines in that women with FPG values lower than 5.6 mmol/L were not included, whereas women with a 2-hour glucose value between 7.8 and 8.5 mmol/L were. Antenatal glycaemic control (therapeutic target HbA1c of 6%) was maintained with nutritional intervention and metformin in two-thirds (73/115 [63.5%]) of the women with only 4/115 (3.5%) requiring insulin therapy. At the postpartum assessment *(t2)*, most of the women 41/51 (80.4%) were breastfeeding exclusively.

### Characteristics of women within the glucose subcategories at *t2*

Glycaemic status was determined by OGTT and HbA1c measurements at the postpartum evaluation *(t2)*. Women who attended the scheduled follow up visit were classified as having either T2DM (7/51 [13.7%]), pre-diabetes (which included women with IFG and/or IGT) (11/51 [21.6%]) or euglycaemia (33/51 [64.7%]). This subcategorization at *t2* was important to accurately identify the high risk T2DM cases to subsequently assess the accuracy of FPG at *t1* to predict T2DM at *t2*. Characteristics of women were similar between these subcategories as noted in Table 2. The low statistical power due to small numbers caution against over-interpretation of results. There was no difference in the mean BMI of women at either booking (T2DM 40 ± 6.9 kg/m^2^; pre-DM 34.5 ± 4.8 kg/m^2^; euglycaemia 38.7 ± 8.6 kg/m^2^) (p = 0.236) or at the postpartum (T2DM 39.2 ± 7.4 kg/m^2^; pre-DM 34.1 ± 3.7 kg/m^2^; euglycaemia 37.5 ± 8.6 kg/m^2^) (p = 0.328) evaluation. The majority of the women within each of the subcategories were classified as World Health Organization (WHO) obese categories 2 or 3 with body weight at *t2* returning to similar values recorded earlier when booking for pregnancy care. Amongst the women classified into the T2DM subcategory at *t2*, more than two thirds (5/7 [71.4%]) had prior overt T2DM and all [7/7 (100%)] required glucose lowering therapy antenatally.

**Table 2:** Comparison of characteristics within the glucose subcategories at *t2*.

	T2DM *(n = 7)*	Pre-DM *(n = 11)*	Euglycaemia *(n = 33)*	p-value
**Age** (years)^a^	32.9 ± 5.4	34.1 ± 3.8	33.5 ± 6.4	n.s
>35 n (%)	3 (42.9%)	4 (36.4%)	17 (51.5%)	-
**Ethnicity** n (%)				
Black African	5 (71.4%)	8 (72.7%)	26 (78.8%)	n.s
Mixed ancestry	1 (14.3%)	3 (27.3%)	5 (15.2%)	-
White	0 (0%)	0 (0%)	0 (0%)	-
Other	1 (14.3%)	0 (0%)	2 (6.1%)	-
**Family history** n (%)				
First degree relative with T2DM	3 (42.9%)	2 (18.2%)	14 (42.4%)	-
**Previous GDM** n (%)	2 (28.6%)	1 (9.1%)	4 (12.1%)	-
**Anthropometry at booking**				
Weight (kg)^a^	100.7 ± 20.6	88.7 ± 11.8	99.9 ± 23.3	n.s
Body mass index (BMI) (kg/m^2^)^a^	40.0 ± 6.9	34.5 ± 4.8	38.7 ± 8.6	n.s
BMI > 40 kg/m^2^ n (%)	3 (42.9%)	2 (18.2%)	17 (51.5%)	-
**Gestation**				
Antenatal diagnosis (weeks) ^a^	22.6 ±9.4	27.1±6.7	29.5 ± 5.3	n.s
**HFDP type** n (%)				
GDM (NICE criteria 2015)	2 (28.6%)	7 (63.6%)	29 (87.9%)	-
Overt DM (IADPSG; WHO 2013)	5 (71.4%)	4 (36.4%)	4 (12.1%)	-
**Antenatal glucose lowering intervention** n (%)				
Lifestyle	0 (0%)	2 (18.2%)	8 (24.2%)	-
Pharmacological therapy	7 (100%)	9 (81.8%)	25 (75.8%)	n.s
**Caesarean section** n (%)				
Elective	2 (28.6%)	1 (9.1%)	10 (30.3%)	-
Emergency	1 (14.3%)	5 (45.5%)	13 (39.4%)	-
**Macrosomia** (>4000 g) n (%)	1 (14.3%)	1 (9.1%)	5 (15.2%)	-
**Postpartum anthropometry** *(t2)*				
Weight (kg) ^a^	99.5 ± 19.3	87.3 ± 10.9	96.3 ± 22.5	n.s
Body mass index (kg/m^2^)^a^	39.2 ± 7.4	34.1 ± 3.7	37.5 ± 8.6	n.s
BMI > 40 kg/m^2^ ^a^	1 (14.3%)	2 (18.2%)	15 (45.5%)	-
**Breastfeeding (exclusive)** *(t2)* n (%)	7 (100%)	9 (81.8%)	27 (81.8%)	n.s

Values are presented as either absolute values (n (%)) or mean ± SD^a^. Statistical analysis: Chi-square test or

^a^ One-Way ANOVA. Due to the low statistical power, no differences could be detected between subgroups. Abbreviations: n.s–not significant.

### Biochemistry

Biochemical evaluation for all time points is summarized in Table 3.

**Table 3:** Biochemical parameters at the different study time points.

Parameters	Total cohort *(n = 115)*	Follow up *(n = 51)*	No follow up *(n = 64)*	p-value
***t0*: Antenatal (at diagnosis)**				
**Glycaemic assessment**				
Fasting glucose (mmol/L)^a^	5.7 (5.1–6.3)	5.7 (5.0–6.3)	5.7 (5.1–6.1)	n.s
2h glucose (mmol/L)^a^	8.8 (7.9–9.6)	8.9 (8.0–9.6)	8.8 (7.9–9.6)	n.s
HbA1c				
• (%)^a^	5.7 (5.3–6.3)	5.7 (5.3–6.3)	5.7 (5.4–6.3)	n.s
• (mmol/mol)^a^	39 (34.4–45.40)	39(34.4–45.4)	39(34.4–45.40)	
**Diagnostic classification** n (%)				
Overt DM (IADPSG; WHO 2013)	25 (21.7)	14 (27.5)	11 (17.2)	n.s
• *HbA1c (≥ 6*.*5%) (≥ 48 mmol/mol)*	*22 (19*.*1)*	*12 (23*.*5)*	*10 (15*.*6)*	n.s
• *Fasting glucose (≥ 7 mmol/L)*	*16 (13*.*9)*	*9 (17*.*6)*	*7 (10*.*9)*	n.s
• *2h glucose (≥ 11*.*1 mmol/L)*	*13 (11*.*3)*	*8 (15*.*7)*	*5 (7*.*8)*	n.s
GDM (NICE criteria 2015)	90 (78.3)	37 (72.5)	53 (82.8)	n.s
• *Fasting glucose (≥ 5*.*6 mmol/L)*	*64 (55*.*7)*	*29 (56*.*9)*	*35 (54*.*7)*	n.s
• *2h glucose (≥ 7*.*8mmol/L)*	*86 (74*.*8)*	*40 (78*.*4)*	*46 (71*.*9)*	n.s
***t1*: Post-delivery (24-72h)**				
**Glycaemic assessment**				
Fasting glucose (mmol/L)^a^				
• Plasma	4.5 (4.0–5.3)	4.5 (4.0–5.0)	4.7 (4.1–5.4)	n.s
• POC	4.8 (4.3–5.4)	4.6 (4.2–5.2)	4.9 (4.3–5.4)	n.s
HbA1c (%)				
• (%)^a^	5.8 (5.5–6.4)	5.9 (5.6–6.4)	5.8 (5.4–6.3)	n.s
• (mmol/mol)^a^	40 (36.6–46.4)	41 (37.7–46.4)	40 (36.6–46.4)	
**Diagnostic classification n(%)**				
T2DM *(FPG ≥ 7mmol/L)*	*2 (1*.*7)*	*0 (0)*	*2 (3*.*1)*	-
Impaired fasting glucose *(6*.*1–6*.*9 mmol/L)*	*3 (2*.*6)*	*1 (2*.*0)*	*2 (3*.*1)*	-
***t2*: Postpartum follow-up (4–12 weeks)**				
**Glycaemic assessment**				
Fasting glucose (mmol/L)^a^				
• Plasma	-	4.7 (4.4–5.3)	-	
• POC	-	4.9 (4.3–5.7)	-	
2h glucose (mmol/L)^a^				
• Plasma	-	6.3 (4.9–8.5)	-	
• POC	-	6.4 (5.0–8.0)	-	
HbA1c				
• (%)^a^	-	5.9 (5.6–6.4)	-	
• (mmol/mol)^a^		41 (37.7–46.4)		
**Diagnostic classification n(%)**				
T2DM	***-***	7 (13.7)	-	
• *Fasting plasma glucose (≥ 7mmol/L)*	-	*4 (7*.*8)*	-	
• *2h plasma glucose (≥ 11*.*1 mmol/L)*	-	*3 (5*.*9)*	-	
• *HbA1c (≥ 6*.*5%) (≥ 48mmol/mol)*	-	*5 (9*.*8)*	-	
Impaired fasting glucose (6.1–6.9 mmol/L)	***-***	2 (3.9)	-	
Impaired glucose tolerance (7.8–11 mmol/L)	***-***	9 (17.6)	-	

Values are presented as either absolute values (n (%)), or median (IQR)^a^. Statistical analysis: Fisher’s exact test or Mann Whitney non-parametric test with two-tailed p-value. No differences were detected between the women that returned for assessment at 4–12 weeks postpartum (n = 51) and those who were lost to follow up (n = 64). Abbreviations: n.s non-significant.

#### In-hospital evaluation following delivery (t1)

The *t1* in-hospital evaluation was performed within 24–72 hours after delivery (mean time-interval of 41 ± 15 hours) and was done after a minimum of 8 hours fasting (mean fasting time 11 ± 2 hours). At *t1*, diabetes sub-classification was based on FPG only. The median FPG at *t1* was 4.5 (4.0–5.3) (IQR) mmol/L and the median POC FG was 4.8 (4.3–5.4) (IQR) mmol/L. Of the 115 participants, 5/155 (4.3%) women had elevated FPG with 2/5 with values in keeping with T2DM (≥ 7 mmol/L) and the remaining 3/5 had impaired fasting glucose (6.1–6.9 mmol/L). An HbA1c of ≥ 6.5% (48 mmol/mol) was documented in 23/115 women at *t1*. A significant correlation (r = 0.920; p<0.001, n = 98 pairs) was evident between the capillary FPG and concomitant POC FG done at *t1* (data not shown).

#### Postpartum evaluation 4–12 weeks following delivery (t2)

**Fig 2:**
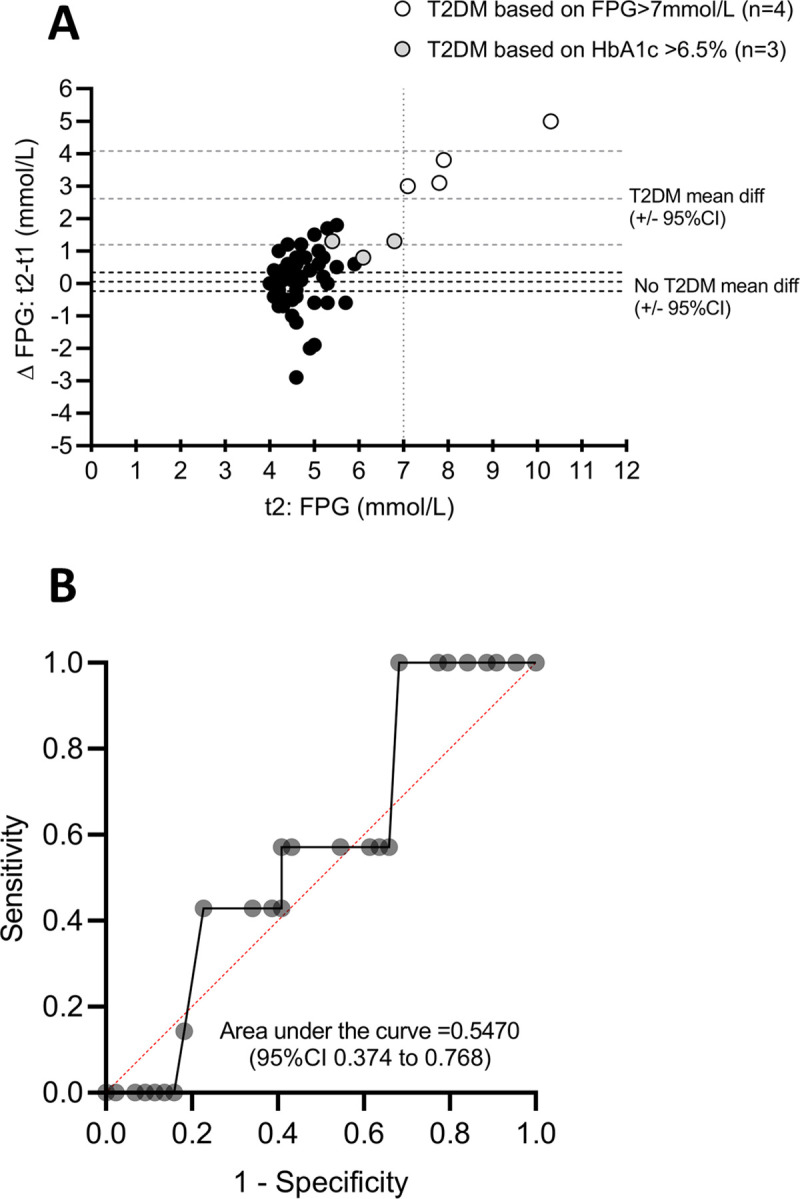
Fasting glucose (mmol/L) levels. A) Bland-Altman graph illustrating the difference in FPG levels at t2 and t1 in women diagnosed with T2DM (n = 7) vs women without T2DM (n = 44) at 4–12 weeks postpartum (t2). B) Receiver Operator Curve of FPG values at 24-72h post-delivery (t1).

Fifty-one women (51/115 [44.3%]) returned for the *t2* postpartum follow-up (mean time interval of 9.1 ± 1.9 weeks). WHO criteria for the diagnosis of diabetes in non-pregnant women was utilised for diagnosis at *t2*. The incidence of T2DM at *t2* was 13.7% (7/51). For these women diagnosed with T2DM, 4/7 were diagnosed based on FPG. In 3/4 of these women the 2-hour glucose value was ≥11.1 mmol/L and in 2/4 the HbA1C values was ≥ 6.5%, while 3/7 women were diagnosed based on HbA1C ≥ 6.5% (48 mmol/mol) only. There was a significant correlation (r = 0.765, p<0.001, n = 51 pairs) between FPG and POC FG at *t2* (data not shown). The concordance of the laboratory vs POC diagnosis of T2DM on OGTT was 100% (4/4) for the fasting glucose values and 66.7% (n = 4/6) for the 2-hour OGTT time point. The difference in the FPG at *t1* and *t2* is illustrated in Fig 2A. The women diagnosed with T2DM at t2 based on FPG (4/7) showed a substantial rise in FPG values from *t1* to *t2* (mean difference 2.614±1.54 mmol/L) whereas the women diagnosed based on HbA1c (3/7) could not be easily distinguished. Although the t1 FPG values can correctly identify the women that do not have T2DM it cannot predict the 4–12 week FPG in the group of women that were diagnosed with T2DM at *t2*. Immediate post-partum T2DM evaluation is thus unreliable. To identify a possible threshold value for FPG at t1 (that is different from T2DM diagnostic criteria) for screening purposes a ROC analysis was performed. Due to the small number of cases, a sensitivity value of >0.9 (90%) only gave specificity of 0.045 (45%) with a non-supportive area under the curve (p = 0.5470). A screening threshold value could thus not be identified (Fig 2B). A power analysis was performed using the data obtained at *t2* to determine the required sample size and indicated that n = 185 patients that return for the postpartum evaluation (t2) is needed to accurately predict a threshold value using ROC. The current data therefore only indicate that T2DM diagnosis cannot be performed within 24-72h post-delivery using FPG>7mmol/L.

#### Comparison of fasting glucose at all time points (t0, t1 and t2)

**Fig 3:**
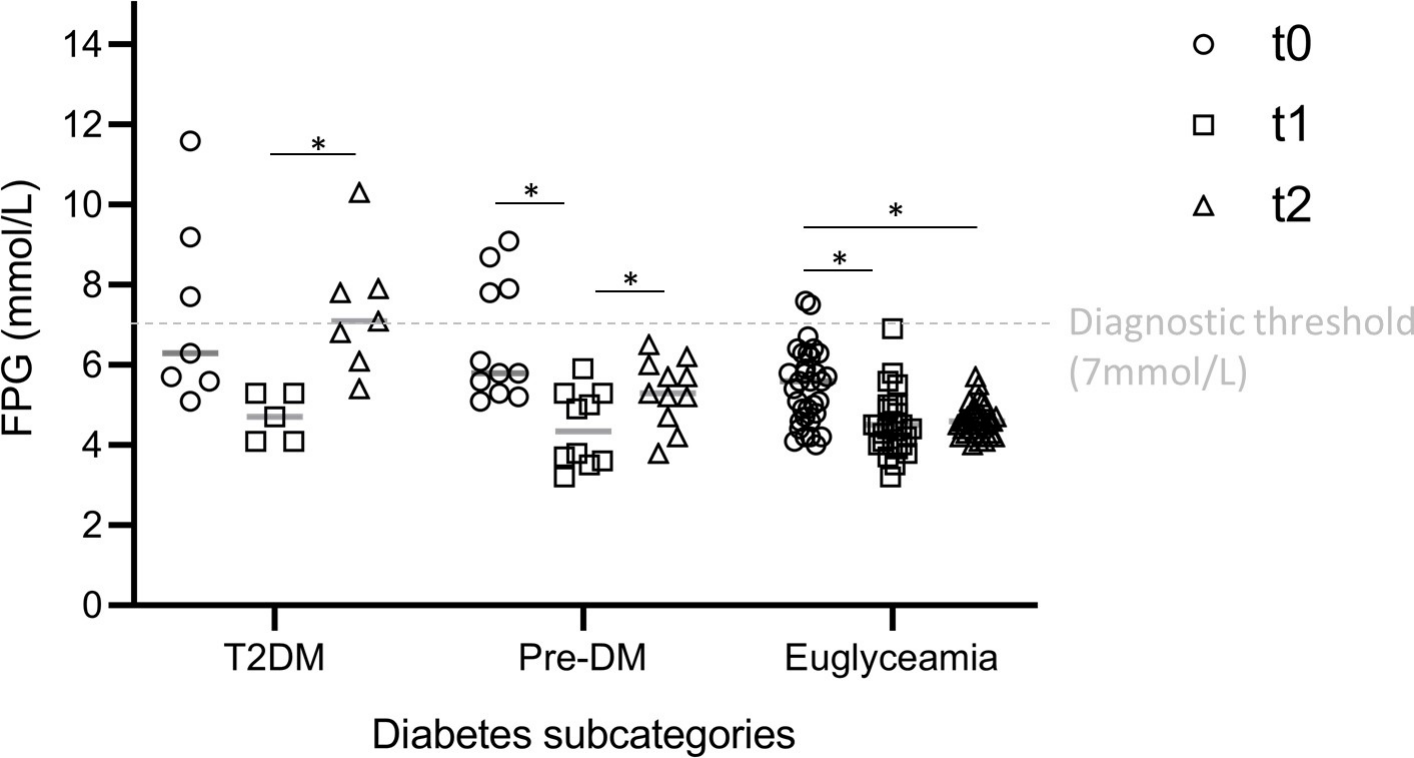
Comparison of fasting plasma glucose levels within the diabetes subcategories at each time point. Values are presented as mmol/L. T2DM (n = 7)–type 2 diabetes; pre-DM (n = 11)–pre-diabetes; euglycaemia (n = 33). t0 –at diagnosis; t1 – 24h to 72h post-delivery; t2–4–12 week postpartum evaluation. Statistical Analysis: Mixed model ANOVA with Tukey post hoc test. *p<0.05 indicate significate effect of time within each diabetes subcategory.

Considering the cohort (n = 51) who returned for the postpartum follow up visit and therefore had glucose evaluations at all 3 time points *(t0*, *t1 and t2)*, FPG values at delivery (*t1*) (4.5 (4.0–5.0)) (95% CI: 4.24 to 4.83) were overall lower compared to *t0* (5.7 (5–6.3)) (95% CI: 5.1 to 5.78) (p = 0.038). A statistically significant decline in FPG values was evident at t1 compared to *t0* for the final pre-diabetes (t1: 4.35 (3.6–5.3)) (95% CI: 3.7 to 5.1) (p = 0.013) and the euglycaemia (t1: 4.5 (4.0–4.97) (95% CI: 4.2 to 4.8) (p<0.001) subcategories. From *t1* to *t2* an increase in FPG concentration was noted in the T2DM (t2: 7.1 (6.1–7.9)) (95% CI: 5.8 to 8.8) (p = 0.040) and pre-diabetes (t2: 5.3 (4.7–6.0)) (95% CI: 4.7 to 5.8) (p = 0.035) subcategories (Fig 3). Forty-three women (43/51 [84.3%]) had paired FPG levels at *t1* and *t2*. There were no significant correlations between FPG at *t1* and FPG at *t2* (r = 0.280; p = 0.070, 43 pairs). POC FG in the 51 women at both time points revealed similar findings (r = 0.115; p = 0.422, 51 pairs).

#### Glucose subcategories in women assessed at all time points (t0, t1and t2)

Of the 51 women who completed glycaemic assessment at all three time points, 14/51 (27.5%) had abnormal glucose levels at *t2*. Of these women, 4/14 had persistent T2DM based on OGTT criteria and an additional 3/14 had an HbA1c ≥ 6.5% along with pre-diabetes values on OGTT. Another 7/14 met OGTT criteria for pre-diabetes. Of the 14/51 women with abnormal glucose assessment at *t2*, not a single individual had an elevated fasting glucose at *t1*.

The women who had overt T2DM at *t0*, all (24/115) had normal fasting glucose levels at *t1* except for one with IFG (1/24). Of these 24 women diagnosed with T2DM at *t0*, 14 returned for the postpartum visit. At *t2* 5/14 had persistent T2DM, 4/14 had pre-diabetes and 5/14 were euglycaemic on OGTT.

## Discussion

The results of this study showed a significant, temporary improvement in maternal fasting glucose in all subcategories of glycaemia 24–72 hours following delivery. In contrast to similar published work, early post-delivery glucose testing was not found to be a feasible alternative to the standard of care 4-12-week postpartum visit in women with HFDP within our population group [14,17–21]. The study failed to identify high risk individuals and did not demonstrate that in-hospital fasting glucose could help to direct resources to those most in need of surveillance. The antenatal characteristics of the 51 (44.3%) women with HFDP who returned for follow-up at 4–12 weeks postpartum were similar to the women who failed to attend.

Routine early postpartum glucose evaluation at 4–12 weeks by means of an OGTT is standard practice in women with HFDP and endorsed by all major diabetes societies world-wide [8,9,10,13]. The postpartum glucose assessment is very important in these women as it not only provides an opportunity to identify women with T2DM, but also allows for timeous interventions to prevent or delay the onset of T2DM for others in this high risk subset.

The prevalence of abnormal glucose homeostasis after HFDP in South Africa is high, with recent reported figures ranging between 40–46% [22] The poor attendance rates of the recommended 4–12 weeks postpartum assessments globally, argues against the convenience of this visit for mothers who prioritize the care of their new-born at this time. This problem is reflected locally with a low retention rate at Tygerberg Hospital of ~30% despite an established electronic reminder strategy directed at all patients with HFDP (reminders via SMS or WhatsApp calls) [15]_._ The ability, therefore, to identify women with HFDP at highest risk for sustained glucose abnormalities, during delivery hospitalization could reduce the need for early postpartum assessment and enable clinicians to direct resources towards those most in need. Pregnancy-related insulin resistance has been shown to resolve within hours after placental delivery [11] Therefore, it is biologically plausible to evaluate glucose status post-partum whilst women are still hospitalized. The present study was done to assess if the gap between outpatient post-partum glucose evaluation and most women who fail to attend this follow up, could be bridged.

Various studies, including some conducted locally, have demonstrated that a fasting plasma glucose (FPG) identifies the majority of patients with GDM [6,22]_._ The utility of FPG as an antenatal screening test for GDM has thus been proposed as an alternative for the cumbersome OGTT. The possibility that FPG assessment during in-hospital stay post-delivery could replace gold standard OGTT assessment at 4–12 weeks postpartum was therefore explored. Published studies pertaining to early post-delivery glucose assessment in GDM women as an alternative to the 4–12 week OGTT were mostly favourable. Curtis et al., (2017) explored this novel approach in 118 GDM women in the UK [14]_._ Similar to the findings of our study 110/115 women (95.6%) had a normal FPG at *t1*. However, no OGTT comparison was available at 4–12 weeks and the assumption of persistent normalization was based on the in-hospital fasting glucose only. Nabuco and colleagues (2016) compared the outcome of an early postpartum (48–72 hours after delivery) OGTT with the standard assessment at 6 weeks. They aimed to determine cut-off glucose values at this time point in order to identify the presence of pre-diabetes and T2DM at 6 weeks [17]_._ The prevalence of diabetes and pre-diabetes in their cohort of 82 women with GDM based on an OGTT performed at 48–72 hours after birth was 3.7% and 32.9%, respectively, whereas the prevalence based on the 6 week OGTT was 8.5% and 20.7%, respectively. These figures are similar to ours at the 6 week postpartum follow-up (13.7% T2DM; 21.5% pre-diabetes), but differ significantly from ours at the earlier time point where our study only identified one patient with a fasting glucose in keeping with pre-diabetes (2%). They determined that a FPG of 4.3 mmol/L and 4.4 mmol/L were the optimal screening cut-off levels post-delivery to identify individuals with pre-diabetes or T2DM at 4–12 weeks postpartum. The investigators propose that these FPG values should prompt clinicians to screen after pregnancies complicated by GDM. What is noteworthy is the low cut-off fasting glucose values post-delivery associated with persistent dysglycaemia at 6 weeks. This indicated that this study also noted lower FPG post-delivery compared to the 6 weeks assessment in keeping with our observation.

In a multicentre study from the United States, Waters et al., (2020) compared the OGTT 2–5 days following delivery with an OGTT at 4–12 weeks in 157 women with GDM [18]_._ They documented normal glucose tolerance in 73 women, pre-diabetes in 74 and overt diabetes in 10 women following delivery. Compared to our findings, more women with abnormal glucose tolerance were identified at *t1*, likely reflecting differences in antenatal glucose status and diagnostic criteria for GDM. None of the 73 normoglycaemic women at *t1* had T2DM at *t2*, however, of the 157 women tested at *t1*, 79 (47.3%) changed diagnostic categories between the two assessments. More than half of the women (54; 57.4%) with abnormal glucose homeostasis at *t1* improved to normalize their glucose homeostasis at *t2* and 18 women (24.7%) progressed from normal at *t1* to pre-diabetes at 4–12 weeks. Based on their data, the authors concluded that a normal OGTT during the delivery hospitalization appears to exclude postpartum DM, but that it remains unclear whether immediate postpartum testing should replace traditional testing for all women with GDM. Carter et al., (2018) also reported that a normal early postpartum glucose assessment carries a very high specificity (100%) and NPV (96.7%) for DM at 6 weeks, albeit with small numbers [19]. Bhalli et al., (2018) evaluated 138 women in Pakistan with GDM [20], by doing an OGTT soon after delivery. They showed that a normal OGTT at 48–72 hours post- delivery rules out DM with a high specificity (96.1%) and negative predictive value (94.1%) for DM [20]. A study from Iran [21] reported that only five of 61 women with normoglycaemia on OGTT during post-delivery hospitalization re-developed dysglycaemia at postpartum follow-up 6–12 weeks later (8.2%). The specificity for OGTT to predict sustained dysglycaemia 70.9%.

Our study had limitations and confounding factors could have influenced the outcome. Glucose homeostasis during post-delivery hospitalisation (*t1*) was only assessed looking at fasting plasma glucose and a more comprehensive assessment of glycaemic status with an OGTT was not performed. We sought a method to assess glycaemic status during post-delivery hospitalization that was practical and feasible within an overburdened and resource-limited obstetric setting. Prior studies in our population have confirmed the ability of a FPG to detect the majority of patients with GDM [6,22], hence the decision to evaluate the usefulness of FPG only. As we assessed fasting glucose values only and did not perform an OGTT, our work is not directly comparable with the previous studies mentioned. Our study population was small (n = 115) and only 44.3% of our cohort returned for the 4–12 week postpartum assessment. Our population size and the return rate, is however not dissimilar to other published work. The antenatal characteristics of women with HFDP who returned for their later postpartum visit did not differ from the women who failed to attend ensuring that the two cohorts were favourably congruent. Due to large patient numbers and pressure on hospital beds, women are often discharged very soon after normal vaginal delivery. This resulted in the inclusion of more women delivered by caesarean section in the cohort. We, however, do not believe that this influenced our ability to compare glycaemic assessments at *t1* and *t2*.

The increased insulin resistance during pregnancy is multifactorial and implicate hormonal changes, molecular mechanisms, exosomes, cytokines and maternal fat mass. Most of these changes are expected to return to normal within hours after delivery. The pathophysiological explanation for the temporary improvement in fasting glucose following delivery in our study cohort remains uncertain. Future studies should attempt to illuminate the underlying pathophysiological mechanisms by evaluating insulin secretion and resistance in women with HFDP to explain the lowered fasting glucose observed at *t1* in this study.

We did not measure maternal weight at *t1*. All women with HFDP at TH are enrolled in an intense lifestyle modification program that attempts to minimize weight gain in overweight, pregnant patients. This may have resulted in weight reduction with improved insulin sensitivity at *t1* that reversed at *t2* due to the fact that weight reduction may potentially not have been maintained.

Many of our patients underwent caesarean sections (78/115). The delivery process itself, as well as the surgical intervention, may be associated with decreased nutrient intake. The initiation of breast feeding increases maternal energy utilization and may also temporarily impact on glucose homeostasis.

## Conclusion

This study found that FPG at 24–72 hours postpartum is not a sensitive indicator of glycaemic status at 4–12 weeks *(t2)*. All 14 women who had an abnormal glucose assessment at *t2* (seven with T2DM, seven with pre-diabetes) had a completely normal glucose assessment at *t1*. In addition, no significant correlation between FPG at *t1* and FPG at *t2* (r = 0.280; p = 0.070) could be demonstrated. Pragmatic solutions for low postpartum return rates in women with HFDP should be pursued. Based on these findings, in-hospital assessment of fasting glucose 24–72 hours postpartum cannot replace the OGTT 4–12 weeks postpartum.

## Supporting information

S1 Data (XLSX)
